# p300 Alters Keratinocyte Cell Growth and Differentiation through Regulation of p21^Waf1/CIP1^

**DOI:** 10.1371/journal.pone.0008369

**Published:** 2010-01-13

**Authors:** Ping-Pui Wong, Adam Pickard, Dennis J. McCance

**Affiliations:** Centre for Cancer Research and Cell Biology, Queen's University Belfast, Belfast, United Kingdom; Roswell Park Cancer Institute, United States of America

## Abstract

**Background:**

p300 functions as a transcriptional co-activator to regulate many cellular responses such as cell growth, transformation, development and differentiation. It has been shown to affect the transcriptional activity of p53 which regulates p21^Waf1/CIP1^ expression, however, the role of p300 in differentiation remains unclear.

**Methodology and Principal Findings:**

Knockdown of p300 protein with short hairpin RNA (shRNA) molecules delays human neonatal foreskin keratinocyte (HFKs) differentiation. Moreover, depletion of p300 increases the proliferative capacity of HFKs, extends the life span of cells and allows differentiated HFKs to re-enter the cell cycle. Studies indicate that depletion of p300 down-regulates the acetylation and expression of p53, and chromatin immunoprecipitation (ChIP) analysis shows that induction of p21^Waf1/CIP1^ in early differentiation is a result of p300 dependent activation of p53 and that depletion of p21^Waf1/CIP1^ results in the delay of differentiation and a phenotype similar to p300 depletion.

**Conclusions:**

p300 has a direct role in the control of cell growth and differentiation in primary epithelial cells, and p21^Waf1/CIP1^ is an important mediator of these p300 functions.

## Introduction

p21^Waf'1/CIP1^ is a member of the Cip/Kip family of cyclin dependent kinase inhibitors that bind to and inhibit the cyclin dependent kinases, cyclinE/cdk2 and cyclinD/cdk4, which in turn prevents the phosphorylation of retinoblastoma protein (Rb) in order to induce cell cycle arrest, cell differentiation or senescence [1]–[5]. The family members (p21^Waf'1/CIP1^,p27^Kip1^ and p57^Kip2^) shares a high degree of sequence homology in their N-terminal domain, which allows them to recognize a wide range of cyclin/cdk targets [6]. In contrast, the C-terminal domain of p21^Waf'1/CIP1^, which can bind and inhibit the DNA replication function of proliferating nuclear antigen (PCNA) is unique [7], [8]. Studies of p21^Waf1/CIP1^ null mouse keratinocytes indicated that the induction of p21^Waf1/CIP1^ in early differentiation is required for initial commitment of keratinocytes to differentiate [9], [10]. However, p21^Waf1/CIP1^ expression has to be down-regulated at a later stage of differentiation, as sustained over-expression of p21^Waf1/CIP1^ inhibits keratinocyte terminal differentiation independently of the cell cycle [11]. A tight regulation of p21^Waf1/CIP1^ expression is therefore required for the process of keratinocyte differentiation [12], [13]. However, the mechanism of control of p21^Waf1/CIP1^ expression during early differentiation is unclear. One regulator is p300, which has been shown to modulate p21^Waf1/CIP1^ promoter activity during mouse keratinocyte differentiation [14] and has also been shown to regulate muscle cell differentiation in a MyoD dependent pathway [15], [16]. p300 is a histone acetyltransferase which functions as a modulator of chromatin structure [17], [18]. During gene transcription, it acetylates the N-terminal tails of the core histone to destabilize nucleosomes, thereby facilitating the binding of transcription factors to DNA [19]. p300 also functions as a transcriptional co-activator and is recruited to promoter regions via direct interaction with various transcription factors [20]. It has been shown to acetylate and regulate the transcriptional activity of p53 and p63 [21]–[23], both of which are upstream regulators of p21^Waf1/CIP1^ [24], [25]. However, the mechanism by which p300 acts to affect cell growth and differentiation of normal human epithelial cells has not been elucidated. We report here that the induction of p21^Waf1/CIP1^ expression in early differentiation is regulated by p53 with p300 involved in p53 activation of p21^Waf1/CIP1^. Knockdown of endogenous p300 by shRNA causes a decrease in expression of differentiation markers of HFKs in organotypic raft culture. It also increases the proliferative capacity of HFKs and allows differentiated cells to re-enter cell cycle, which is also observed in p21^Waf1/CIP1^ deficient cells. Moreover, exogenous expression of p21^Waf1CIP1^ rescues the expression of differentiation marker in p300 depleted cells. Taken together, our results indicate that p300 has a direct role in the control of cell growth and differentiation in primary epithelial cells and that p21^Waf1/CIP1^ is an important mediator of these p300 functions.

## Results

### Knockdown of p300 Inhibits Early Keratinocyte Differentiation

**Figure 1:**
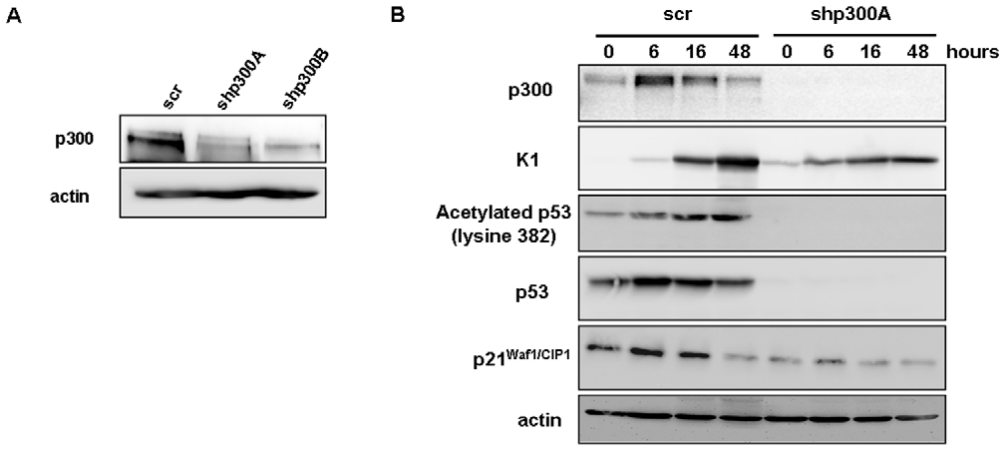
Stable p300 depletion inhibits differentiation. HFKs were induced to differentiate in CaCl_2_ medium and harvested at the indicated time points (t = 0, 6, 16, 48 hrs). (**A**) Western blot analysis of levels of p300 in control and p300 depleted HFKs using scrambled shRNA (scr) two shRNA molecules (shp300A and shp300B) directed against p300. (**B**) Cells stably expressing p300 targeting shRNAs were induced to differentiate and harvested at the indicated time points. Western blot analysis indicates that knocking down p300 dramatically reduces the p300 mediated acetylation and expression of p53. There is also a decrease in p21^Waf1/CIP1^ and K1 levels in p300 depleted HFKs. The results shown are representative of three independent experiments.

**Figure 2:**
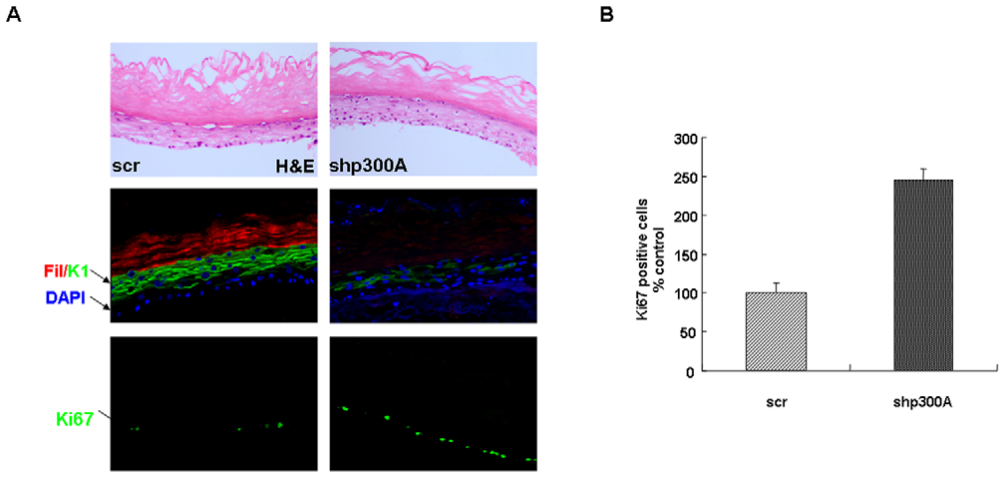
p300 has a role in proliferation and differentiation of organotypic keratinocyte raft cultures. (**A**) H&E staining of organotypic raft cultures of control (scr) and p300 knock down (shp300A) cells (upper panel). Immunostaining of raft sections indicates that level of early marker keratin 1 (K1) and late marker filaggrin (Fil) are significantly reduced in knockdown cells (middle panel). Ki67 immunostaining reveals that there is an increase in the number of proliferative cells in the basal layer of p300 knockdown rafts compared to control (lower panel). (**B**) Graph represents percentage of Ki67 positive cells in p300 depleted cells, expressed relative to scrambled control (Mean +/− SE, two independent biological replicates).

To study the role of p300 in HFK differentiation, we knocked down endogenous p300 by using two retrovirally expressed shRNA molecules directed against p300 and a scrambled control. Western blot analysis indicated that levels of p300 were decreased significantly in both p300 knockdown lines (∼80%) (Figure 1A). Scramble and shp300 cell lines were then treated with calcium to induce differentiation and harvested at indicated time points. In control cells p300 dependent acetylation of p53 on K382 was observed, but this is absent in p300 depleted cells due to the significant reduction in p53 protein levels (Figure 1A). These experiments were carried out 3 times with different batches of HFKs and we always observed a decrease in p53 protein levels in p300 depleted cells, although mRNA levels were unchanged compared to control cells (Figure S1). Depletion of p300 also reduces the expression of early differentiation marker, keratin 1 (K1) and p21^Waf1/CIP1^ during calcium treatment (Figure 1B). We have only shown results with one of the shRNA molecules as the outcome with the other was similar. Reduced levels of induction of differentiation markers was also observed in organotypic raft cultures, where knockdown of p300 resulted in reduced expression of K1 (early marker) and filaggrin (late marker) (Figure 2A). There was also an increase in proliferation of HFKs in the basal layer of the raft cultures in the knockdown cells (Figure 2A and B). Moreover, we transiently knocked down the expression of p300 or CBP with short interfering RNA (siRNA) molecules and induced the cells to undergo differentiation. Strikingly, depletion of p300, but not CBP, down-regulated the p300 dependent p53 protein levels, which in turn reduced the expression of p21^Waf1/CIP1^ and K1 in differentiating keratinocytes (Figure S2). Since p300 depleted cells expressed normal levels of CBP in differentiation, these results suggest that p300, but not CBP, plays a role in keratinocyte differentiation.

### Knockdown of p300 Increases Life Span of Keratinocytes and Results in Cell Cycle Re-Entry during Differentiation

**Figure 3:**
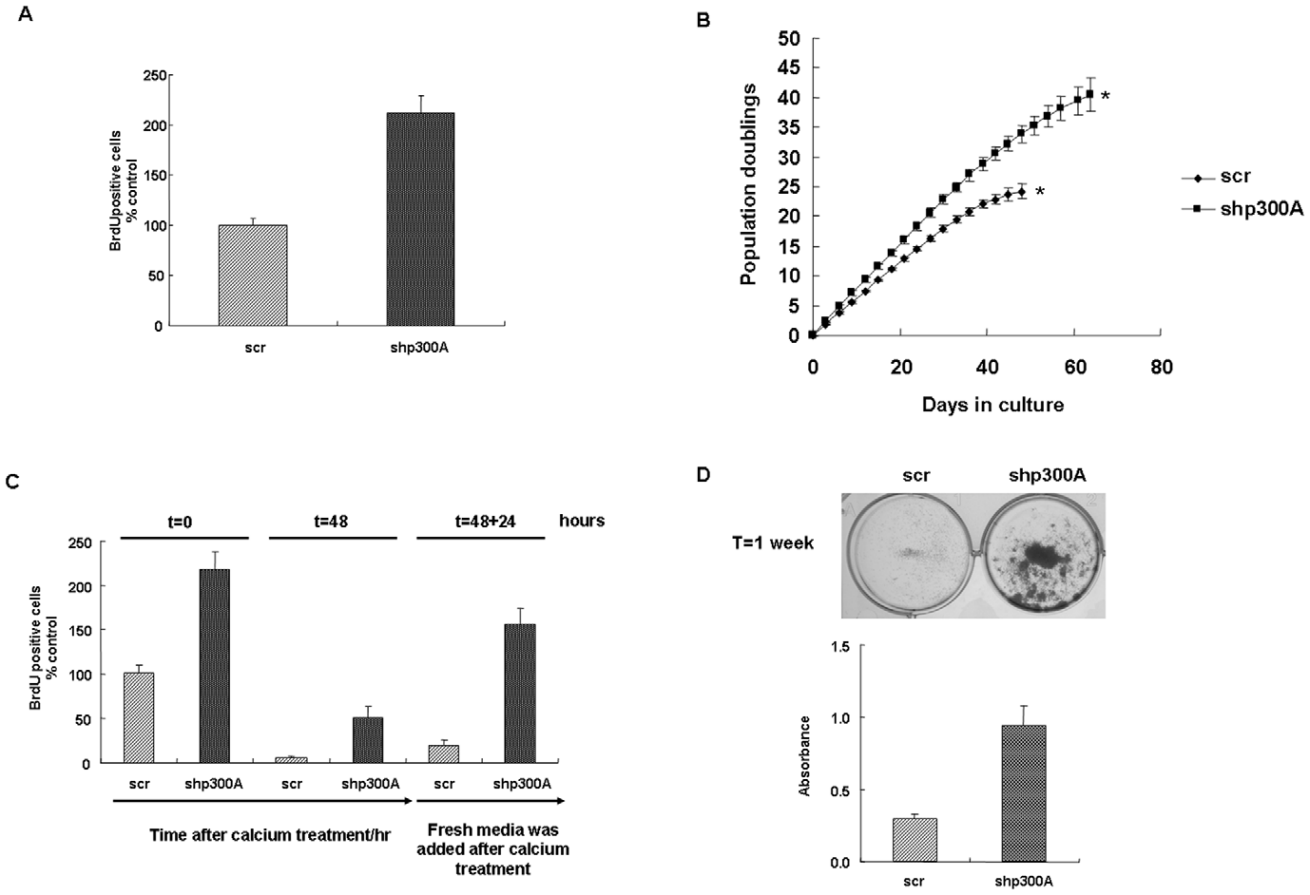
Stable p300 knockdown increases the proliferation of HFKs and allows the differentiating HFKs to re-enter cell cycle. (**A**) Cycling HFKs stably expressing p300 targeted shRNAs in culture were pulsed with BrdU prior to fixation. Graph represents percentage of BrdU positive cells, expressed relative to scrambled control in cycling cells (mean +/− SE of four independent experiments). (**B**) Graph shows life span progression of stably transduced cell lines. Asterisk (*) indicates the population doubling (PD) level at which senescence occurred. (**C**) Stably transduced cell lines were seeded onto coverslips and treated with 1.5 mM calcium for 48 hours. After 48 hours, cells were incubated with fresh media for 24 hours (t = 48+24). (**D**) Differentiated HFKs were replated onto six well plates and cells were stained with crystal violet after incubating with fresh media for 7 days. Histogram represents three independent experiments calculating the average A540 nm reading of crystal violet solutions following destaining of colonies.

To further study the effects of p300 on cell proliferation we investigated firstly, the effects on proliferation in cycling cells and secondly, if there was an increase in the life span of p300 depleted cells. There was an increase in bromodeoxyuridine (BrdU) uptake in p300 depleted cycling cell (Figure 3A) and also a significant increase in life span, with depleted cells going through senescence after 40 population doublings as opposed to control cells which went though senescence around 25 population doublings (Figure 3B). This result is an average of three experiments using different batches of keratinocytes.

Since p300 depletion causes inhibition of differentiation and increased proliferation of cells we reasoned that it may also allow cells to escape from the permanent cell cycle arrest that is required for terminal differentiation of keratinocytes. p300 depleted cells were calcium treated to induce differentiation and after 48 hours the cells were placed in growth medium to determine the capability to re-enter the cell cycle as measured by BrdU uptake. Approximately 25% of depleted cells did not completely exit the cell cycle after 48 hours in calcium while all control cells had exited the cycle (Figure 3C). When after 48 hours in differentiation medium, cells were transferred to growth medium for 24 hours, many p300 depleted cells re-entered the cell cycle compared to control cells (Figure 3C, t = 48+24). To determine if the cells that re-enter the cycle are viable, we replated cells in growth medium and allowed the cells to grow for 7 days. A significant proportion of the p300 depleted cells, but not the control cells, that re-entered the cycle were viable and grew into colonies (Figure 3D).

### p300 Regulates p21^Waf1/CIP1^ Promoter Activity during Differentiation

**Figure 4:**
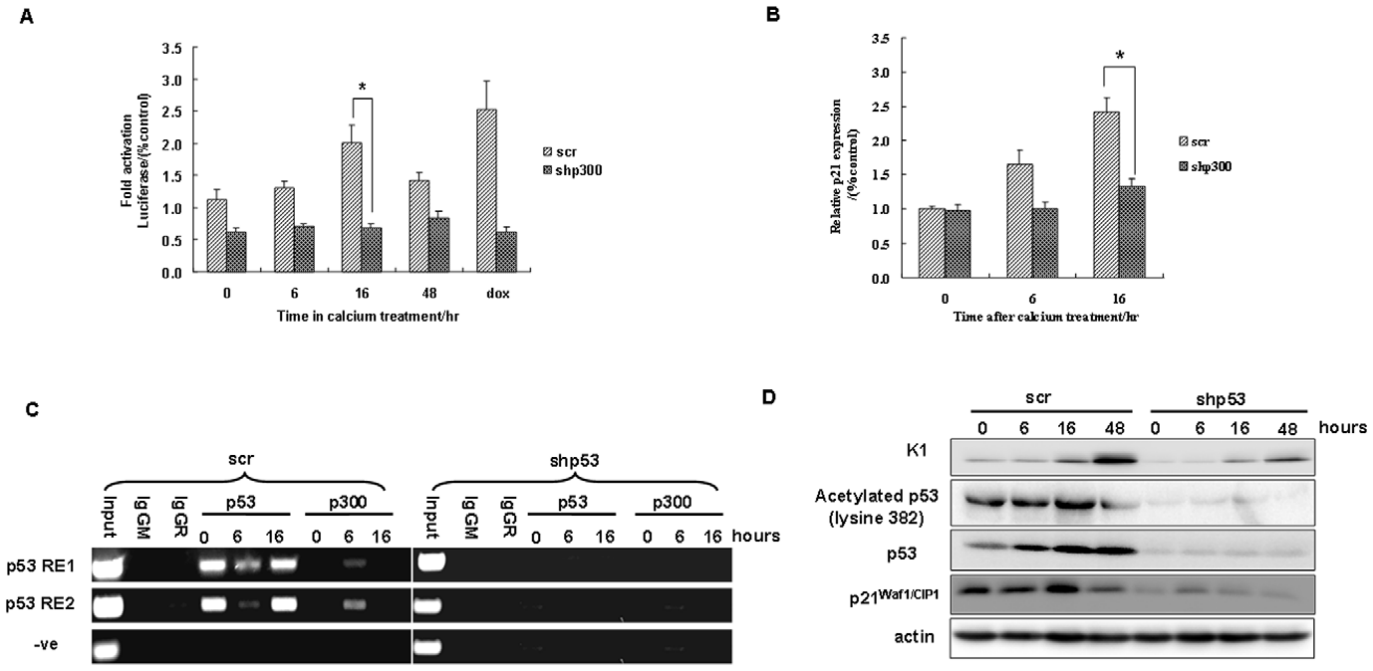
p300 regulates p21^Waf1/CIP1^ expression at transcriptional level. Stably expressing p300 targeted shRNAs cells were transfected with p21^Waf1/CIP1^ promoter luciferase construct. Transfected cells were either kept in low calcium (0.5 mM) or high calcium (1.5 mM) for the indicated times prior to the termination of the experiment (72 hours after transfection). Doxorubicin (0.1 µg/ml) was used as a positive control in this experiment. (A) Depletion of p300 down-regulates p21^Waf1/CIP1^ promoter activity after 16 hours of calcium treatment (Mean +/− SE, three independent biological replicates; asterisk (*) p<0.01 relative to relevant control, Student's t test). (B) Real time quantification confirms that depletion of p300 inhibits the transcription of p21^Waf1/CIP1^ during differentiation (Mean +/− SE, two independent biological replicated; asterisk (*) p<0.05 relative to relevant control, Student's t test). (C) ChIP assay for p53 and p300 binding to two p53 response elements located in the p21^Waf1/CIP1^ promoter in HFKs induced to differentiate by addition of calcium. PCR analysis of DNA precipitated by a p300 or p53 antibodies indicates that p300 only interacts with both p53 response elements within the p21^Waf1/CIP1^ promoter after 6 hours calcium treatment. Left panel: p53 binding to the p53 respond elements (RE1 and RE2) increases over a 16 hour periods. Right panel: Depletion of p53 results absence of p53 and p300 at both p53 response sites. Mouse (IgGM) and Rabbit IgG (IgGR) are included as negative control. (D) Western blot of the proteins from the same extracts as in (C).

To try and understand the mechanism by which p300 depletion causes an increase in proliferation, we determined whether p300 regulation of p21^Waf1/CIP1^ expression was important. Scramble and shp300 expressing cells were transiently transfected with a pGL-3 plasmid carrying the human p21^Waf1/CIP1^ promoter and cells were either kept in normal calcium medium (60 µM) or exposed to high calcium concentrations (1.5 mM) for the indicated time points. Luciferase assays showed that the knockdown of p300 down-regulated p21^Waf1/CIP1^ promoter activity during keratinocyte differentiation compared to that of the scramble control (Figure 4A). Also, real-time RT-PCR confirms that depletion of p300 significantly reduces p21^Waf1/CIP1^ mRNA during keratinocyte differentiation (Figure 4B), indicating that p300 is important for transcriptional regulation of p21^Waf1/CIP1^ during differentiation. Others have shown that the two p53 binding sites are important for p21^Waf1/CIP1^ transcription and we have found the same requirement in differentiating keratinocytes (Figure S3).

Since p300 can bind and acetylate p53 to activate p21^Waf1/CIP1^ expression during DNA damage and p300 and p53 are required for p21^Waf1/CIP1^ transcription, we studied the ability of p300 to bind p53 response elements in differentiating cells by using chromatin immunoprecipitations. Keratinocyte DNA was PCR amplified with primers specific for sequences that flank the two p53 response elements (RE1 and RE2). As a control for non-specific binding during immunoprecipitation, cross-linked lysates were immunoprecipitated with mouse or rabbit monoclonal IgG antibodies. ChIP analysis revealed that p53 bound strongly to RE1 and RE2 when cells were confluent (t = 0) and persisted during differentiation (Figure 4C). Unlike p53, p300 bound to both RE1 and RE2 only during differentiation (Figure 4C). To confirm that p300 requires p53 to bind to the p21^Waf1/CIP1^ promoter, we stably knocked down the expression of p53 in HFK by using retroviral vector (shp53) and Western blot analysis confirmed that the level of p53 was significantly reduced (∼80%) (Fig. 4D). Interestingly, knock-down of p53 also reduced the expression of p21^Waf1/CIP1^ during early differentiation (Fig. 4D). ChIP analysis indicated that depleting p53 inhibited p300 binding to both RE1 and RE2 during differentiation (Figure 4C). These results suggest that induction of p21^Waf1/CIP1^ in early differentiation is regulated by p300 dependent activation of p53.

### p21^Waf1/CIP1^ Has a Critical Role in Early Keratinocyte Differentiation

**Figure 5:**
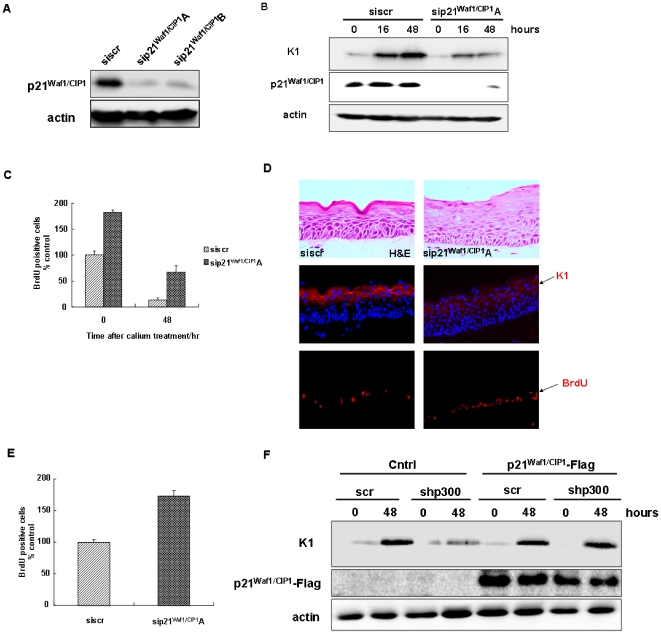
p21^Waf1/CIP1^ knockdown inhibits early keratinocyte differentiation of organotypic keratinocyte raft cultures. HFKs were transiently tranfected with p21^Waf1/Cip1^ targeting siRNA molecules. (**A**) Immunoblotting of the whole cell lysates with p21^Waf'1/CIP1^ antibody shows that both siRNA molecules, sip21^Waf'1/CIP1^A and sip21^Waf'1/CIP1^B, were able to knockdown p21^Waf'1/CIP1^ protein levels efficiently (∼90%) (**B**) Western blot shows p21^Waf1/CIP1^ depletion with sip21^Waf1/CIP1^ A and a decreased in K1 levels after 48 hours of differentiation. (**C**) Transfected cells were seeded onto coverslips, incubated with 1.5 mM calcium for 48 hours, and were pulsed with BrdU prior to fixation. BrdU uptake indicates a 90% increased proliferation of p21^Waf1/CIP1^ knockdown cells prior to the addition differentiation (t = 0). After 48 hours calcium treatment, around 40% of the p21^Waf1/CIP1^ knock-down cells remained BrdU positive. (**D**) H&E staining shows an increase in thickness of epithelia in p21^Waf1/CIP1^ depleted cells (upper panel). Immunohistochemisty staining of organotypic raft cultures indicates that the differentiation marker K1 is reduced in p21^Waf1/CIP1^ knockdown rafts with low levels of K1 (middle panel). Rafts were pulsed with BrdU for 16 hours prior to harvest and BrdU positive cells counted (lower panel). BrdU positive cells were observed in most parabasal cells. (**E**) Graph represents BrdU uptake expressed as percentage of scrambled control (Mean +/− SE, two independent experiments). (F) Stably expressing p300 targeted shRNA cells were transiently transfected with either 1 µg pMT5-p21^Waf1/CIP1^-Flag vector or pCMV empty vector (negative control) for 24 hours. Transfected cells were then incubated with calcium for 48 hours. Western blot analysis indicates that exogenous expression of p21^Waf1/CIP1^ rescued the expression of K1 in p300 depleted cells.

Earlier work suggests that p21^Waf1/Cip1^ has a dual role in mouse keratinocyte proliferation and differentiation [12]. To determine the role in HFKs, cells were transfected with two siRNA molecules targeting p21^Waf1/CIP1^ and Western blotting of whole cell lysates with p21^Waf'1/CIP1^ antibody shows that both siRNA molecules, sip21^Waf'1/CIP1^A and sip21^Waf'1/CIP1^B, were able to knockdown p21^Waf'1/CIP1^ protein levels efficiently (∼90%) (Figure 5A). Transfected cells were induced to differentiate at indicated times and harvested for immunoblotting analysis, which showed that the protein level of K1 was significantly down-regulated in the p21^Waf'1/CIP1^ depleted cells (Figure 5B). Also, p21^Waf1/CIP1^ depleted cells were more proliferative than control cells as measured by BrdU uptake and a significant number of these cells remained BrdU positive 48 hours after differentiation induced by calcium treatment (Figure 5C). HFKs depleted of p21^Waf1/CIP1^ were used to generate organotypic raft cultures and harvested 5 days after lifting to the air-liquid interface. Depletion of p21^Waf1/CIP1^ disrupts the formation of granular and cornified layers in the epidermis with reduced K1 expression and increased proliferation in the basal layer, resulting in a thicker epithelium (Figure 5D). Depletion of p21^Waf1/CIP1^ increased the number of BrdU positive cells by 70% in the basal layer compared to that of the control (Figure 5D & E). Interestingly, BrdU positive cells were also observed in the suprabasal layer of p21^WAF1/CIP1^ depleted raft, indicating that these cells did not exit cell cycle properly as they migrate upwards. To determine whether exogenous expression of p21^Waf1/CIP1^ can rescue the proliferation and differentiation defect of p300 depleted cells, we transiently transfected the scramble and shp300 cell lines with either the vector containing human p21^Waf1/CIP1^ cDNA with a N-terminal Flag tag or pCMV empty vector as a negative control and the cells induced to differentiate for 48 hours. Western blotting indicates that exogenous expression of p21^Waf1/CIP1^ restored the expression of K1 in p300 depleted cells (Figure 5F) and reduced proliferation to the level in control cells (Figure S4). Taken together, our results indicate that p300 may regulate keratinocyte cell growth and differentiation through regulation of p21^Waf1/CIP1^.

## Discussion

We have shown that p300 is an important component for human keratinocyte differentiation and that the ability to regulate p21^Waf1/CIP1^ during the early phase of keratinocyte differentiation is one of the key roles. Depletion of p300 or p21^Waf1/CIP1^ has similar phenotypes with increased proliferation and reduced expression of differentiation markers. In cells depleted of p300, the level of p53 protein falls significantly, suggesting that p300 is required for p53 stability, since mRNA levels are the same as in control cells. This reduction in p53 protein levels was a consistent finding in primary keratinocytes depleted of p300. In cells depleted of CBP, levels of p53 remained similar to control cells, indicating that the effect on p53 levels was p300 specific. In depleted p300 cells, p21^Waf1/CIP1^ levels are ***also*** greatly reduced suggesting that p53 is responsible for the increase in p21^Waf1/CIP1^ observed early in differentiation. While previous work had shown that p300 plays a key role in C2C12 muscle cell proliferation and differentiation [26], no knockdown studies were carried out although surprisingly inhibiting p300 acetyltransferase activity did not have an effect on p21^Waf1/CIP1^ levels. In another study, micro-injection of p300 antibody inhibited C2C12 cell differentiation [15]. Since p300 has been shown to regulate G1/S phase transition [27], we asked whether a defect in cycle arrest due to p300 depletion could explain the observed inhibition of differentiation. BrdU incorporation and replating experiments show that differentiated p300 knock-down cells were able to re-enter cell cycle while control cells could not. In addition, p300 depleted cells re-entered the cell cycle and proliferated when transferred from differentiation medium to growth medium. This suggests that p300 plays a critical role in differentiation associated cell cycle arrest. Similar findings were observed by Dotto et al. (1995), who showed that over-expression of p300 can overcome the negative effect of adenovirus E1A on p21^Waf1/CIP1^ promoter in differentiation. Since p300 is a transcriptional coactivator, it requires an interacting protein to bind the p21^Waf1/CIP1^ promoter [18]. We have shown that the p53 response elements of p21^Waf1/CIP1^ promoter were required for transcriptional activation during early differentiation and we also showed that knocking down p300 reduced the levels of p53 during differentiation. Since acetylation of p53 has been recently shown to regulate its stability and transcriptional activity [28], [29], this suggests that acetylation of p53 by p300 may be important for its function in differentiation. However, since we did not show that the acetylation function of p300 is required for ^p21Waf1/CIP1^ transcription and keratinocyte differentiation, the acetylation by p300 is yet to be resolved. However, our ChIP analysis result indicates that p300 interacts with both p53 response elements in differentiating cells, whereas this interaction was inhibited by stably knocking down p53. Also, knocking down p53 caused reduction in p21^Waf1/CIP1^ expression after calcium treatment. A similar finding was observed by Yugawa et al. (2007), who showed that inactivation of p53 by shRNA resulted in repression of keratinocyte differentiation marker and p21^Waf1/CIP1^ expression [30]. Taken together, these results suggest that p300 regulates p21^Waf1/CIP1^ promoter in a p53 dependent pathway. However, Missero et al. (1995) showed that induction of p21^Waf1/CIP1^ was observed in p53 null mouse keratinocyte cell during differentiation [14]. This may be explained by the chronic absence of p53 is somehow compensated for by other p53 family members, whereas acute depletion has a more immediate effect on p21^Waf1/CIP1^ levels. Moreover, we show that p63 has a dual role in keratinocyte growth and differentiation, partially via regulation of p21^Waf1/CIP1^ (unpublished data). This raises a possibility that p300 may regulate p63 transcriptional activity to control p21^Waf1/CIP1^ expression in keratinocyte differentiation [22]. In conclusion, our findings show that p300 and p21^Waf1/CIP1^ are required for differentiation of human keratinocytes and that p21^Waf1/CIP1^ is regulated by p53.

## Materials and Methods

### Cell Culture, Infections and Transfections

Primary human foreskin keratinocytes (HFKs) were harvested from neonatal foreskins, cultured in low calcium in Epilife (Casade) and transduced with retrovirus produced in ΦNYX packaging cell line (ATCC) as previously described [31]. The retroviral vectors (scramble, shp300, and shp53) used in this study were purchased from Origene Technologies, U.S.A. For calcium induced differentiation, confluent monolayers of HFKs were induced to differentiate by withdrawal of growth factors and addition of 1.5 mM CaCl_2_. Differentiation of HFK cell lines in organotypic raft cultures was carried out as previously described for transduced lines [32]. The raft cultures were harvested, fixed in 4% PFA and then embedded in paraffin for subsequent sectioning and staining with haematoxylin and eosin (H&E). To label cells synthesizing DNA, cultures were pulsed with 10 µM bromodeoxyridine (BrdU) for 20 minutes prior to fixation. For the raft culture, 20 µM BrdU was added to the media 16 hours prior to harvest. For siRNA transfection, complexes are produced by combining 100 pmols of siRNA with 100 µl optimen (Invitrogen) followed by the addition of 3 µl FuGeneHD and incubated at room temperature for 15 minutes prior to addition to cells at a final concentration of 100 nM. Cells were incubated with siRNA/FugeneHD complexes for 6 hours. The sequences of p21^Waf1/CIP1^ siRNA molecules were previously described [33], [34]. siGENOME SMART pool p300 and CBP siRNA molecules were purchased from Dharmacon.

### Luciferase Assays

HFKs were transiently transfected with the p21^Waf1/Cip1^ luciferase reporter constructs as previously described [14]. p21^Waf1/Cip1^ promoter deletion luciferase reporter constructs (0-luc to 4-luc) were kindly provided by Dr. Wafik S. El-Deiry [35]. All these constructs were sub-cloned into the XbaI site of pGL-3 basic (Promega), except the 0-luc that was inserted into a blunt-ended Xho1 site of pGL3-basic. p21^Waf1/Cip1^-mut1 luciferase report vector construct was kindly provided by Professor Wei-Guo Zhu [36]. pMT5-p21^Waf1/CIP1^-Flag over-expression vector was purchased from Addgene. All transfections were performed with Fugene 6 and cells were harvested to 72 hours after transfection. Luciferase measurements were performed by using the dual-luciferase kit (Promega).

### Real-Time RT-PCR Analysis

RNA extraction was carried out with High Pure RNA isolation kit (Roche) according to manufacturer's instructions. RNA (1 µg) was treated with RQ1 RNAase free DNase (Promega) prior to first stand cDNA synthesis using random primers with transcriptor high fidelity cDNA synthesis Kit (Roche) according to manufacturers instructions. Amplification of PCR products was quantified using FastStart SYBR Green Master (Roche) according to manufacturers instruction and fluorescence montoried on a DNA Engine Peltier Thermal Cycler (Bio-Rad) equipped with a Chromo4 Real-Time PCR Detection System (Bio-Rad) and melting curve analysis also performed. In brief cDNA samples were diluted 1∶10 and quantified by amplification against serial dilutions of appropriate control cDNA with the following cycling conditions: initial denaturation 95°C for 10 minutes, 40 cycles of 95°C-15 seconds, 58°C-15 seconds, 60°C-60 seconds. Expression levels were assessed in triplicate, normalized to large ribosomal protein (RPLPO) levels and graphs represents the combined results of two independent biological replicates. The specific primers for this analysis were as follows: p21^Waf1/CIP1^ 5′ AGCGGAACAAGGAGTCAG 3′ (sense) and 5′ CGTTAGTGCCAGGAAAGAC 3′ (antisense); p53 5′ CCAGGGCAGCTACGGTTTC′3 (sense) and 5′ CTCCGTCATGTGCTGTGACTG′3 (antisense) [37].

### Chromatin Immunopreciptation (ChIP) Assays

ChIP assays were carried out using ∼2×10^6^ keratinocytes fixed with 1.5% formaldehyde for 20 minutes. Cells were washed twice in ice cold PBS and suspended in collection buffer (100 mM Tris-HCl pH 9.4, DTT 10 mM Protease inhibitors). They were then subsequently washed with 1 mL of NCP1 buffer (10 mM EDTA, 0.5 mM EGTA, 10 mM Hepes pH 6.5, 0.25% Triton X-100) and NCP2 buffer (1 mM EDTA, 0.5 mM EGTA, 10 mM Hepes pH 6.5, 200 mM NaCl) to isolate nuclei. Cells were then spun down and resuspended in 1 mL sodium dodecyl sulfate lysis buffer (0.5% Empigen BB, 1% sodium dodecyl sulfate, 10 mM EDTA, 50 mM Tris-HCL pH 8.0) supplemented with complete protease inhibitor cocktail (Roche), and DNA in the cross-linked preparations was sonicated 6 times for 20 seconds each, to an average fragment size of 500 bp using Sonics Virba Cells Sonicator (24% amplitude) (Sonics & Materials Inc. USA). The insoluble material was removed by centrifugation, and soluble chromatin samples were incubated overnight at 4°C with 2 µg of monoclonal antibodies against p53 (clone DO-1; Santa Cruz Biotechnology), p300 (clone C20; Santa Cruz Biotechnology) or control immunoglobulin G (IgG) and 25 µl of pre-blocked Dynabeads Protein G (Invitrogen), made up to 1 ml final volume with IP buffer (1% Triton X-100, 0.1% sodium deoxycholate, 10 mM EDTA, 50 mM Tris-HCL pH 8.0). The next day, the immune complexes were washed eight times with 1 mL of RIPA buffer (50 mM Hepes pH 8.0, 1 mM EDTA pH 8.0, 1% NP40, 0.7% Deoxycholate, 0.5 M LiCl, Protease inhibitor). Afterwards, the complexes were resuspended in 1 mL of 1X TE buffer and spun down at 3000 rpm for 3 minutes. The samples were then eluted with 50 µl of elution buffer (10 mM Tris pH 8.0, 1 mM EDTA, 10% SDS) at 65°C for 10 minutes vortexing every two minutes during incubation. The eluted samples were spun for 30 second at 15000 rpm and then transferred to fresh tubes. The elution process was repeated and 70 ul elution buffer added. Input templates were purified from 5% of the original lysates in parallel with the eluted immunoprecipitated samples. Cross-linking was reversed by incubating the samples at 65°C for 16 hours. The samples were then purified by using a Qiagen DNA purification kit following the manufacturer's protocol. The recovered DNA (4 µl from 30 µl immunoprecipitated chromatin DNA or 1 µl from the 60 µl input DNA control) was subjected to PCR amplification using GoTaq green Felix PCR reaction kit. The specific primers for this analysis were used as previously described [25], [38], [39].

### Replicative Life Span Determination

Cells were plated at 2×10^5^ cells per p60 dish in Epilife media, refed every 2 days, and subcultured 3 days after plating, before growth was slowed by high cell density. Population doublings (PD) per passage was calculated as log_2_(number of cells at time of subculture/number of cells plated). Cumulative PD was plotted against total time in culture to determine replicative life span and the onset of senescence.

### Western Blot Analysis

Protein lysate concentrations were 50 µg for western blots as previously described [40]. The following primary antibodies were used in this study: mouse monoclonal anti-p53 (DO-1 Santa Cruz Biotechnology 1∶1000), mouse monoclonal anti-p21^Waf1/Cip1^ (BD Phamingen 1∶1000), mouse monoclonal anti-p300 (1B1 Abnova 1∶500), rabbit polyclonal anti-acetylated p53 (lysine 382, Cell signaling 1∶1000), rabbit polyclonal anti-keratin 1 (Convance 1∶2500), mouse monoclonal anti-Flag (Sigma Aldrich, 1::2000) and mouse monoclonal anti-β-Actin (Sigma 1∶10000). Luminescence was determined by incubation with Chemiluminescence Detection Reagent (PerkinElmer, USA) and exposed using FluorChem SP imaging system (Alpha Innotech, USA).

### Immunofluorescent Staining

Tissue embedding and haematoxylin and eosin staining were performed by standard techniques. Images were taken on an Olympus BH-2 microscope with an Olympus D25 camera using Cell B software (Olympus). Five micrometer thick paraffin-embedded sections were deparaffinized with xylene, rehydrated with a series of step down concentration of ethanol and antigen retrieval carried out with boiling 1x antigen retrieval buffer (BioGenex, San Ramon, CA). Briefly, sections were permeabilised with 10%FCS/0.2% Trition X-100 for 30 minutes at room temperature, rinsed and incubated with primary antibody overnight at 4 in 10% FCS. The next day, the sections were washed and incubated with secondary antibody at room temperature for 1 hour, washed and mounted with Prolong Gold antifade reagent plus DAPI (Molecular Probes). The following antibodies were used in this study: mouse monoclonal BrdU (BD Pharmingen 1∶100), mouse monoclonal anti-filaggrin (Neomarkers 1∶50 no retrieval), rabbit polyclonal anti-ki67 (Santa Cruz Biotechnology 1∶100), rabbit polyclonal anti-keratin 1 (Covance 1∶2500 no retrieval), and goat anti-mouse and anti-rabbit secondary conjugated to Alexafluor 488 nm or 594 nm (Molecular Probes 1∶400). BrdU pulsed cells on coverslips were fixed for 15 minutes with 4% formaldehyde, washed 3x with PBS, submitted to antigen retrieval and stained as described above. For raft sections, the number of Ki67 or BrdU positive cells was counted for a minimum of 10 fields of view. For coverslips, a minimum of 10 fields of view and 500 cells were counted on each slide and expressed as percentage of BrdU positive cells (BrdU/DAPI). In all cases, immunofluorescent images were captured on a Leica AF6000 inverted fluorescence microscope and Leica AF imaging software. Exposure times were kept constant within an experiment.

## Supporting Information

Figure S1 Depletion of p300 does not affect the p53 expression at transcriptional level during keratinocyte differentiation. Stably expressing p300 targeted shRNAs cells were incubated with calcium for the indicated times courses. Real time quantification indicates that p300 knock-down does not affect the expression of p53 at transcriptional level during keratinocyte differentiation.
(0.04 MB TIF)

Figure S2 p300 knock-down, but not CBP, inhibits early keratinocyte differentiation of organotypic raft. HFKs were transiently tranfected with either p300 or CBP targeting siRNA molecules. (A and B) Western blots indicate that depletion of p300, but not CBP, reduces the p300 mediated acetylation and expression of p53, and causes decreased expression of p21^Waf1/CIP1^ and K1 in p300 depleted differentiating HFKs. (C) H&E staining shows an increase in thickness of epithelia in p300 depleted cells (upper panel). Immunohistochemisty staining of organotypic raft cultures indicates that the differentiation markers K1 is reduced in p300 knock-down rafts only (middle panel). Rafts were pulsed with BrdU for 16 hours prior to harvest and BrdU positive cells counted (lower panel). BrdU immunostaining reveals that there is an increase in the number of proliferative cells in the basal layer of p300 knock down rafts compared to control or CBP (lower panel). (D) Graph represents BrdU uptake expressed as percentage of scrambled control (mean +/− SE, two independent experiments).
(0.49 MB TIF)

Figure S3 The p53 response element of p21^Waf1/CIP1^ promoter is required for its activity in differentiation. (A) Schematic representing reporter vectors used in panels B and C. For the p21^Waf1/CIP1^-mut1 reporter vector, the distal p53 binding site (RE1) was altered by site-direct mutagenesis to GAAAC. (B) HFKs cells were transfected with constructs shown in panel A. Loss of distal p53 response element inhibits the activation of p21^Waf1/CIP1^ promoter in differentiating cells. (C) HFKs were transfected with a mutated p21^Waf1/CIP1^ promoter luciferase reporter vector (p21^Waf1/CIP1^-mut1). Mutation of the distal p53 response element (RE1) abrogates the induction of p21^Waf1/CIP1^ promoter activity during differentiation. All luciferase assays were normalized for transfection efficiency with a renilla reporter vector (mean +/− SE, three independent biological replicated; asterisk (*) p<0.05 relative to relevant control, Student's t test).
(0.20 MB TIF)

Figure S4 Over-expression of p21^Waf1/CIP1^ rescues the increase in proliferation in p300 depleted cells. Stably expressing p300 targeted shRNA cells were transiently transfected with either 1 µg pMT5-p21^Waf1/CIP1^-Flag vector or pCMV empty vector (negative control) for 24 hours. Transfected cells were then incubated with calcium for 48 hours. BrDU incorporation in control and p21^Waf1/CIP1^ transfected cells was assessed after 48 hours transfection. Expression of p21^Waf1/CIP1^ reduced the number of proliferating cells.
(0.07 MB TIF)
