# Supplementary figures and images for: p300 Alters Keratinocyte Cell Growth and Differentiation through Regulation of p21Waf1/CIP1
Source: PLoS One. 2010 Jan 13;5(1):e8369. doi: 10.1371/journal.pone.0008369 (PMC2805707; doi:10.1371/journal.pone.0008369)

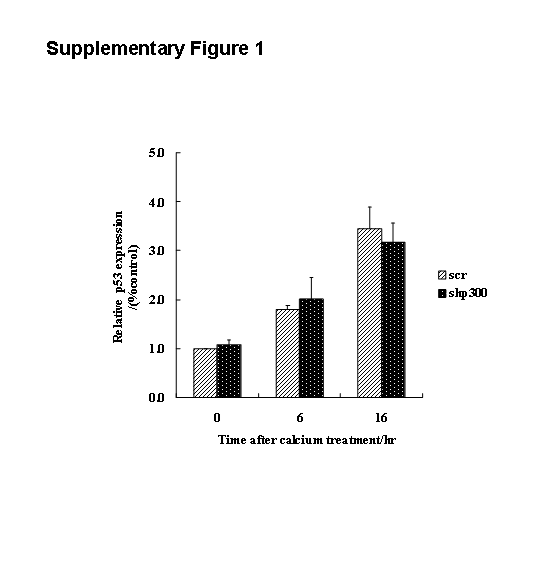

Supplement: Figure S1 — Depletion of p300 does not affect the p53 expression at transcriptional level during keratinocyte differentiation. Stably expressing p300 targeted shRNAs cells were incubated with calcium for the indicated times courses. Real time quantification indicates that p300 knock-down does not affect the expression of p53 at transcriptional level during keratinocyte differentiation. (0.04 MB TIF) [file pone.0008369.s001.tif]

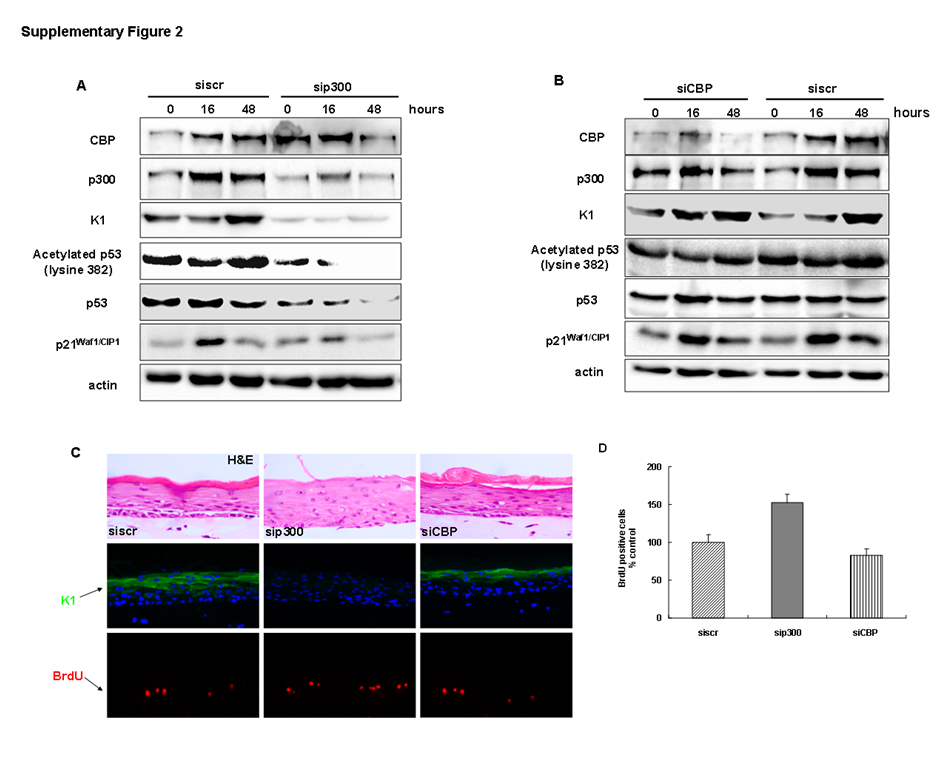

Supplement: Figure S2 — p300 knock-down, but not CBP, inhibits early keratinocyte differentiation of organotypic raft. HFKs were transiently tranfected with either p300 or CBP targeting siRNA molecules. (A and B) Western blots indicate that depletion of p300, but not CBP, reduces the p300 mediated acetylation and expression of p53, and causes decreased expression of p21Waf1/CIP1 and K1 in p300 depleted differentiating HFKs. (C) H&E staining shows an increase in thickness of epithelia in p300 depleted cells (upper panel). Immunohistochemisty staining of organotypic raft cultures indicates that the differentiation markers K1 is reduced in p300 knock-down rafts only (middle panel). Rafts were pulsed with BrdU for 16 hours prior to harvest and BrdU positive cells counted (lower panel). BrdU immunostaining reveals that there is an increase in the number of proliferative cells in the basal layer of p300 knock down rafts compared to control or CBP (lower panel). (D) Graph represents BrdU uptake expressed as percentage of scrambled control (mean +/− SE, two independent experiments). (0.49 MB TIF) [file pone.0008369.s002.tif]

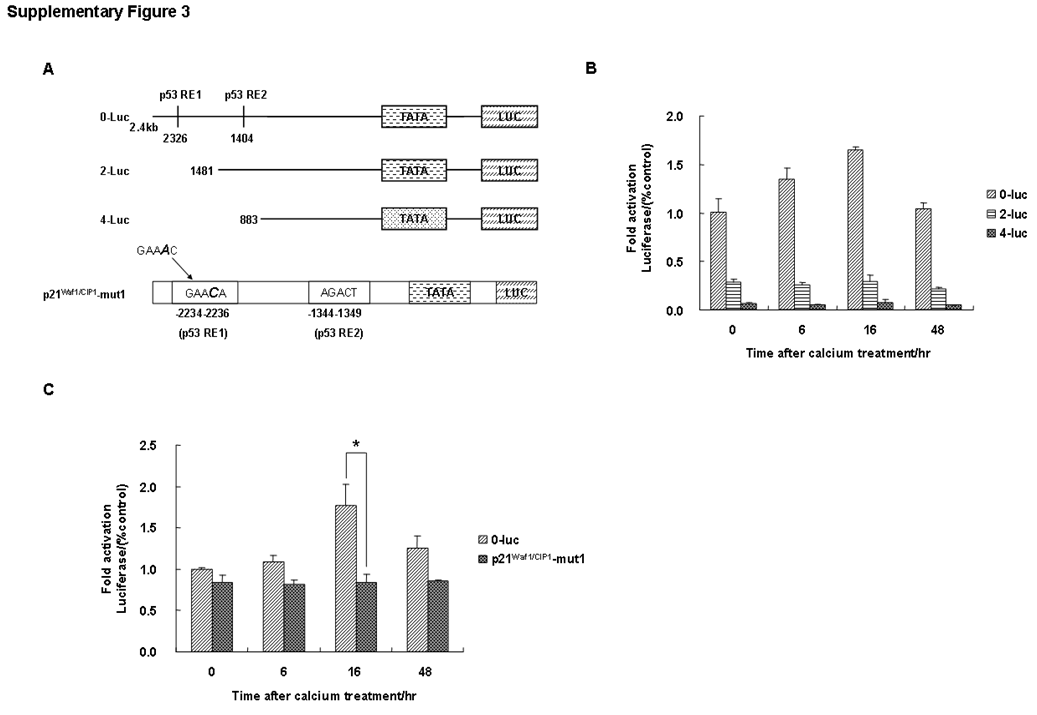

Supplement: Figure S3 — The p53 response element of p21Waf1/CIP1 promoter is required for its activity in differentiation. (A) Schematic representing reporter vectors used in panels B and C. For the p21Waf1/CIP1-mut1 reporter vector, the distal p53 binding site (RE1) was altered by site-direct mutagenesis to GAAAC. (B) HFKs cells were transfected with constructs shown in panel A. Loss of distal p53 response element inhibits the activation of p21Waf1/CIP1 promoter in differentiating cells. (C) HFKs were transfected with a mutated p21Waf1/CIP1 promoter luciferase reporter vector (p21Waf1/CIP1-mut1). Mutation of the distal p53 response element (RE1) abrogates the induction of p21Waf1/CIP1 promoter activity during differentiation. All luciferase assays were normalized for transfection efficiency with a renilla reporter vector (mean +/− SE, three independent biological replicated; asterisk (*) p<0.05 relative to relevant control, Student's t test). (0.20 MB TIF) [file pone.0008369.s003.tif]

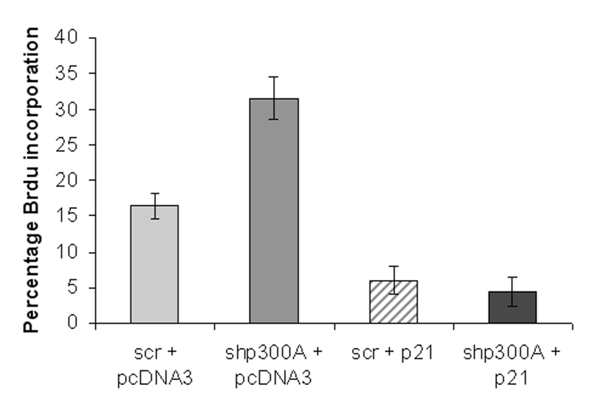

Supplement: Figure S4 — Over-expression of p21Waf1/CIP1 rescues the increase in proliferation in p300 depleted cells. Stably expressing p300 targeted shRNA cells were transiently transfected with either 1 µg pMT5-p21Waf1/CIP1-Flag vector or pCMV empty vector (negative control) for 24 hours. Transfected cells were then incubated with calcium for 48 hours. BrDU incorporation in control and p21Waf1/CIP1 transfected cells was assessed after 48 hours transfection. Expression of p21Waf1/CIP1 reduced the number of proliferating cells. (0.07 MB TIF) [file pone.0008369.s004.tif]
